# There Is Always Something New

**Published:** 1896-04

**Authors:** 


### THERE IS ALWAYS SOMETHING NEW.

One of Boston's leading horsewomen says that driving will grow more popular every day with the coming woman.

Examinations for meat-inspectors to the Department of Animal Industry were held in New York City on April 6, 1896.

Walrus whiskers are removed from these animals, and after drying are forwarded to China, where they are sold for use as toothpicks.

The Esquimaux give the doctor his fee as soon as he comes. If the patient recovers he keeps it, otherwise he returns it to the family.

Canine practice is booming in the city of Washington, and in these days of depressed equine returns forms a valuable contingent to veterinarians practising in large cities.

A double spleen was recently found in the carcass of a hog at one of the abattoirs in St. Louis, Mo. They were normal in size and appearance, and were connected by adipose tissue.

The Courts of Philadelphia have been asked to appoint a receiver for the Provident Mutual Live-stock Insurance Company.

The Veterinary Department of the Iowa Agricultural College, at Ames, has no less than seventeen of her graduates holding positions as instructors, inspectors, State veterinarians, etc.

The veterinarians of Allegheny County should assemble and carefully go over the list of registered veterinarians in that county, and where deaths, removals, etc., have occurred they should present to the prothonotary a properly certified list of these changes, that the register of the county be kept in accordance with the intent of the law. Many of the registries are a disgrace and a dishonor to the calling, and cast a severe reflection on the community.

Aluminum shoes grow in favor among the Russians, where experiments have been made for some time.

The canning of horse-meat at Portland, Oregon, continues to thrive. The consignment of a large number of horses from Utah is under consideration. Feeding-experiments to improve the flesh are being tried.

The January *Journal of Medicine and Science*, of Portland, Me., gives proper and just credit to Prof. Koch for the inestimable gift he has conferred upon the human race in the discovery of tuberculin, in that he has thus produced a perfect diagnostic agent for tuberculosis in the bovine species, by which disease may thus be detected and eliminated, and directly contributing no little to man, by thus removing one of the great danger-sources.

The plans for the construction of an arena, with a seating capacity of eight to ten thousand, in a central location at Philadelphia, are nearing completion. It will be used for large indoor exhibitions, horse, dog, poultry, and pigeon shows, circus purposes, conventions, etc. A riding-school will be attached, and rings for exhibition, with bicycle races, etc. The estimated cost is a half-million of dollars.

Efforts have recently been made to resuscitate the Philadelphia Veterinary Association, which has been wholly inactive for nearly a year.

Bellevue Hospital, New York, has among her patients one Connor, suffering from anthrax, which first seemed only an abscess of the neck. He was formerly a drover, but disclaims any connection with cattle for the past three years. The suggestion is made that he may have been inoculated long ago, the germs lying dormant in his system until recently.

*Harper's Weekly* of February 22 has an interesting article on ponies and pony-breeding.

The sending of eight percheron stallions from Wayne, Illinois, to Belgium, looks like "sending coals to Newcastle."

The English standard of height for polo-ponies has been raised to 14.2 by the Hurlingham Club. The chief motive for increasing the height was to admit better-bred animals of thoroughbred and Arab breeding, a reason which does not hold good in America, where we use the wiry little "cow-ponies of the plains."

Paris, France, leads off in the establishment of Turkish baths for horses and dogs.

An unusually large number of special prizes are offered at the Philadelphia Kennel Show, which will tend to stimulate the rivalry for better-bred dogs of the several species.

